# Comment on “Genomic Hypomethylation in the Human Germline Associates with Selective Structural Mutability in the Human Genome”

**DOI:** 10.1371/journal.pgen.1003332

**Published:** 2013-02-28

**Authors:** Corey T. Watson, Paras Garg, Andrew J. Sharp

**Affiliations:** 1 Department of Psychiatry, Icahn School of Medicine at Mount Sinai, New York, New York, United States of America; 2 Department of Genetics and Genomic Sciences, Icahn School of Medicine at Mount Sinai, New York, New York, United States of America; HudsonAlpha Institute for Biotechnology, United States of America

Copy number variants (CNVs) are a dynamic feature of the human genome that play important roles in human adaptation and susceptibility to both common and rare disease [1]. The distribution of CNVs in mammalian genomes is nonrandom, and several sequence features have been associated with CNV breakpoints and regions of high structural mutability [2]–[8]. Based on an analysis of DNA methylation patterns in human sperm, Li et al. recently reported a significant relationship between CNVs and hypomethylation in the male germline [9], leading to the suggestion that DNA hypomethylation plays a causative role in the generation of structural variation. Given the potentially profound implications of this report for the study of human disease, we read the findings of Li et al. with great interest. However, after systematically reanalyzing the relationship between CNVs and DNA methylation patterns in sperm, we have identified several cryptic confounders in the data that we believe seriously undermine the conclusions of Li et al. We outline and discuss each of these in detail below.

In their analysis, Li et al. first divided the genome into 100 kb windows, with each window being scored for the presence of CNVs ascertained from studies of both normal controls and individuals with a variety of disease states. They then applied two independent methods to estimate germline DNA methylation within each window: (i) directly using published whole genome 15× bisulfite sequencing of sperm DNA [10] and a second low coverage 2.5× dataset, and (ii) indirectly by calculating a Methylation Index (MI) based on the relative occurrence of C>T SNPs defined by the HapMap project [11]. Li et al. then defined windows that showed the lowest mean methylation levels by bisulfite sequencing (either the 1^st^ or 5^th^ percentiles) or had a MI = 0 as “methylation deserts,” and observed an increased prevalence of CNVs in these regions.

We replicated the analysis of Li et al. by dividing the hg18 genome into 100 kb windows and annotating these with several CNV datasets (Table S1) [7], [12]–[14]. We obtained published 15× sperm bisulfite sequencing data [10], which we used for all subsequent analysis of germline methylation levels. Although retained in the analysis of Li et al., we discarded the Y chromosome due to its small size, highly unusual sequence content, and almost complete lack of HapMap SNPs.

We first investigated repeat content within “methylation deserts” identified by Li et al. We observed a strong enrichment for common repeats in windows with the lowest 1% methylation (Figure 1a), with 30% of windows defined as “methylation deserts” containing >99^th^ percentile of total repeat content. In particular, we noted a massive enrichment for satellite repeats within these “methylation deserts.” Satellites comprise 16.6% of sequence in hypomethylated windows, compared to only 0.26% in the rest of the genome, corresponding to a 64-fold enrichment (*p* = 1.4×10^−29^, Mann-Whitney Rank Sum Test). Importantly, as noted by Molaro et al. [10], the vast majority of pericentromeric satellites and many other subtypes of common repeat are hypomethylated specifically in sperm. Due to their repetitive and nonunique nature, pericentromeres and regions with extreme repeat content are also hotspots for structural variation [15], [16], representing a strong confounder in any analysis of CNVs and hypomethylation. Indeed, after removing all 100 kb windows that contain satellites or contain >99^th^ percentile by LINE, SINE, LTR, or total repeat content, we observed that in every dataset analyzed, enrichments for CNVs in windows with the lowest 1% mean methylation either significantly diminished or disappeared completely (Figure 1b).

**Figure 1:**
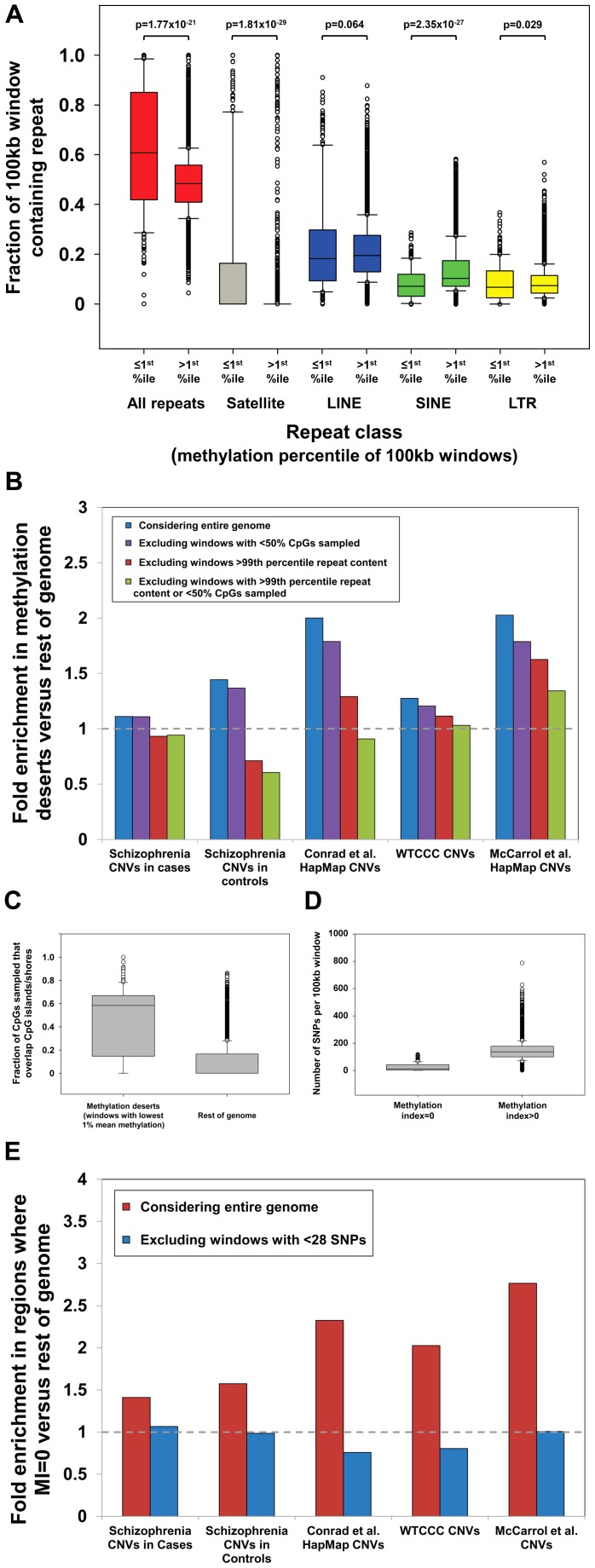
Multiple strong confounders contribute to artifactual associations between CNVs and hypomethylation. (a) Hypomethylated regions of the human genome are highly enriched for satellite repeats. We observed a strong enrichment for satellite repeats in regions of the genome <1^st^ percentile of mean methylation level. Satellites comprise a mean of 16.6% of the hypomethylated windows, compared to only 0.26% in the rest of the genome (∼64-fold enrichment, *p* = 1.4×10^−29^, Mann-Whitney Rank Sum Test). Previous analysis has shown that satellites tend to be strongly hypomethylated in human sperm [10]. Furthermore, given their highly repetitive and dynamic nature, loci rich in satellites are enriched for CNVs (51.7% of windows containing satellites overlap HapMap CNVs [7] compared to 20.5% in the rest of the genome), creating an inherent confounder between CNVs and hypomethylation. (b) No enrichment for CNVs in hypomethylated regions after removal of confounding genomic features. Li et al. reported significant enrichments for overlap with multiple CNV datasets in “methylation deserts” (those with the lowest 1% mean methylation) and regions of the genome with MI = 0 [9]. However, after excluding regions of extreme repeat content (all windows containing satellite repeats, and those >99^th^ percentile by LINE, SINE, LTR, and total repeat content, *n* = 1,716), and/or windows in which only a minority of CpGs were sampled (*n* = 430), all reported CNV enrichments reduce significantly and in most cases disappear entirely. Dashed grey line represents equal prevalence of CNVs between hypomethylated regions compared with the rest of the genome. (c) Bisulfite reads within “methylation deserts” preferentially map to CpG islands/shores. We observed that windows scored as “methylation deserts” by Li et al. (those with the lowest 1% mean methylation) show a strong bias for bisulfite reads to be mapped within ±2 kb of CGIs. As CGIs, especially those associated with the promoters of expressed genes, are typically unmethylated, this creates an underestimate of the mean methylation value in the wider region. Data shown represent fraction of CpGs per window with at least one overlapping read that map within ±2 kb of CGIs, after first excluding all windows containing satellite repeats, or those >99^th^ percentile based on LINE, SINE, LTR, or total repeat content. (d) A huge reduction in SNP density in windows with MI = 0. We observed a massively reduced density of HapMap SNPs in windows with MI = 0 (mean, 25; median, 13) compared to the genome average (mean, 143; median, 137). As mSNPs represent only 8.2% of all SNPs in the genome and the formula used by Li et al. to calculate MI reports MI = 0 when no mSNPs are present, the use of a methylation index based on SNP content is inherently biased to score windows containing only a small number of SNPs as MI = 0. Because of stringent quality filtering, ∼98% of HapMap SNP assays map uniquely within the genome [20]. Therefore, a significant negative correlation exists between SNP density and segmental duplications (*r* = −0.337, *p*<10^−323^), a fraction of the genome that is highly enriched for structural variation [2], [3], [7]. (e) No enrichment for CNVs in regions with MI = 0 after removal of windows with low SNP density. Li et al. reported that windows with MI = 0 are enriched for CNVs identified in several different studies [9]. However, power calculations (Figure S4) show that at least 28 SNPs per window are required to achieve a <10% false discovery rate for MI = 0. After excluding windows containing <28 SNPs (*n* = 811), all enrichments for CNVs in the remaining regions with MI = 0 disappear, indicating that the conclusions of Li et al. are likely artifactual resulting from low SNP density in many CNV regions.

We next considered the influence of problems associated with mapping reduced-complexity bisulfite reads in duplicated regions of the genome. Specifically, we hypothesized that in CNV regions that are often comprised of nonunique sequence, the analysis of Li et al., which only considered uniquely mappable reads, might suffer from significant bias. Consistent with our hypothesis we observed a reduction in the proportion of CpG dinucleotides that had at least one overlapping read in regions defined as “methylation deserts” (Figure S1). As these “methylation deserts” are enriched >2-fold for CpG islands (CGIs) compared to the genome average [9], we measured the distribution of mapped reads across the genome in relation to CGIs and observed a strong tendency for preferential sampling of sites located within CGIs and their flanks (so-called “CpG shores”) in these “methylation deserts” (Figure 1c). Excluding windows containing extreme repeat content, on average 47.5% of CpG dinucleotides sampled by bisulfite sequencing in the remaining “methylation deserts” lie within ±2 kb of CGIs, while in the rest of the genome this figure is only 9.0% (a 5.3-fold enrichment). As the majority of CGIs are unmethylated in sperm [10], [17], any window in which bisulfite reads map preferentially within CGIs will tend to yield artifactual underestimates of the true methylation level in that region.

An illustrative example is shown in Figure S2. This window was classified by Li et al. as a “methylation desert” due to a mean methylation level <1^st^ percentile. However, analysis of the bisulfite sequencing data shows that in fact only 27/355 (8%) of the CpG dinucleotides in this region have been sampled, most of which lie within a CpG island that spans the promoter of *RPS17*, a gene highly expressed in testes [18]. While methylation levels within this CGI are uniformly low, most CpGs in the rest of the window have high methylation levels (>75%), suggesting that the low mean methylation level in this window is not a true reflection of the wider region, and instead results from heavily biased sampling of sites within the CGI. The low frequency of uniquely mapped bisulfite reads in this region is attributable to the presence of a copy number variable segmental duplication of 99.85% identity.

Overall, 80/285 (28%) of the windows defined by Li et al. as “methylation deserts” were >95^th^ percentile based on the fraction of CpGs sampled that lie within ±2 kb of CGIs. Therefore, preferential sampling of CGIs, regions that tend to be inherently unmethylated in sperm [10], [17], likely underlies a significant fraction of the regions labeled as “methylation deserts.” Crucially, after excluding CpGs lying within 2 kb of CGIs, the mean methylation level for the remainder of these windows is in fact *greater* than that in the rest of the genome (79.1% versus 77.2%). These same windows that show biased sampling of CGIs are also enriched for CNVs and genes highly expressed in testes (Figure S3), creating a strong confounder in any attempt to associate hypomethylation with structural variation. Indeed, after excluding 100 kb windows showing extreme preferential sampling of CGIs, in every dataset analyzed enrichments for CNVs in “methylation deserts” significantly diminished or disappeared completely (Figure 1b).

In addition to the use of bisulfite sequencing for measuring DNA methylation, Li et al. also calculated a SNP-based methylation index (MI). The MI is based on the notion that methylated cytosines have an increased vulnerability to transition mutation via spontaneous deamination [19]. Thus, by measuring the relative occurrence of C>T SNPs within CpG dinucleotides (termed “mSNPs”), it is possible to draw inferences about the ancestral methylation state of a region [11]. However, SNP-based studies of structural variation are often compromised due to the fact that many CNV regions show significantly reduced SNP density compared to the genome average (median density of HapMap SNPs within HapMap CNVs [7] is 1 per 1,087 bp, compared to 1 per 738 bp genome-wide). This stems largely from the fact that ∼98% of HapMap SNP assays map uniquely within the genome [20], resulting in markedly reduced SNP density in duplicated portions of the genome, precisely those regions that are also enriched for CNVs [2], [3], [7]. As a result, there is a strong confounding relationship between CNV regions and low SNP density that renders the use of a SNP-based MI inherently flawed for studies of structural variation.

This fact is of particular concern as Li et al. calculated their MI using an equation in which the numerator is the number of mSNPs in each region, and claimed that windows with MI = 0 represent “methylation deserts.” In total, analyzing ∼4 million HapMap SNPs, 8.2% of which are mSNPs, they defined ∼1.5% of the genome as MI = 0. However, because of the formula used, if the number of total SNPs per 100 kb window is low, then the probability of observing no mSNPs, and thus the likelihood that the MI will be zero simply due to insufficient SNP sampling, becomes large. Indicative of this bias in their data, we found that windows with MI = 0 contained a median of just 13 SNPs, compared to a median of 137 in the rest of the genome (>10-fold difference, *p* = 3.2×10^−6^, Mann-Whitney Rank Sum Test; Figure 1d).We performed power calculations based on the relative prevalence of mSNPs in the genome, showing that under a random distribution a minimum of 28 SNPs are required per window to provide <10% false discovery rate for regions with MI = 0 (Figure S4). Only 156/443 (35%) of the windows with MI = 0 identified by Li et al. contain ≥28 SNPs, suggesting that the majority of windows with MI = 0 are false positives simply due to insufficient SNP data. In fact, although Li et al. did not consider windows completely lacking SNPs in their analysis, 123/443 (28%) of regions labeled as MI = 0 contain just 1, 2, or 3 SNPs. Given the almost complete absence of data on which to base this conclusion, we suggest that it would be appropriate to remove such regions from analysis rather than concluding these represent “methylation deserts.” Indeed, after removing windows with insufficient SNP density (*n*<28), we observe that all enrichments for CNVs in regions with MI = 0 vanish (Figure 1e). Li et al. also observed that two-thirds of regions with MI = 0 actually showed high mean methylation levels in sperm by bisulfite sequencing and suggested that this discrepancy might be explained by low methylation specifically in the female germline. Given the problems associated with calculating MI in SNP-poor regions of the genome, we suggest that a more parsimonious explanation would be that many of these regions identified as having MI = 0 are actually false positives resulting from a failure to filter those with low SNP density.

Finally we believe that the approach used by Li et al. in which the genome was first partitioned into 100 kb intervals before associating windows containing CNVs with average methylation levels is poorly suited to address the question in mind, suffering from low resolution and an increased susceptibility to artifacts. Taking a more direct approach, we used published 15× sperm bisulfite sequencing data [10] to calculate mean methylation per base both within and flanking 5,360 nonredundant HapMap CNVs <20 kb in size (mean CNV size 3,789 bp) (Figure 2a) [7]. Although we observed a small decrease in methylation levels within CNVs compared to flanking regions, overall CNV regions have consistently high levels of methylation (mean 69%) that are only slightly lower than the genome average (70%). Furthermore this slight dip in CNV methylation corresponds precisely with an increase in CpG density and an enrichment for CGIs within CNVs (CGIs comprise 1.1% of CNVs compared to 0.75% genome-wide, a 1.4-fold difference; Figure 2b). As most CGIs are unmethylated in sperm [10], [17], this fact alone is likely to account for the small overall reduction in methylation levels associated with CNVs.

**Figure 2:**
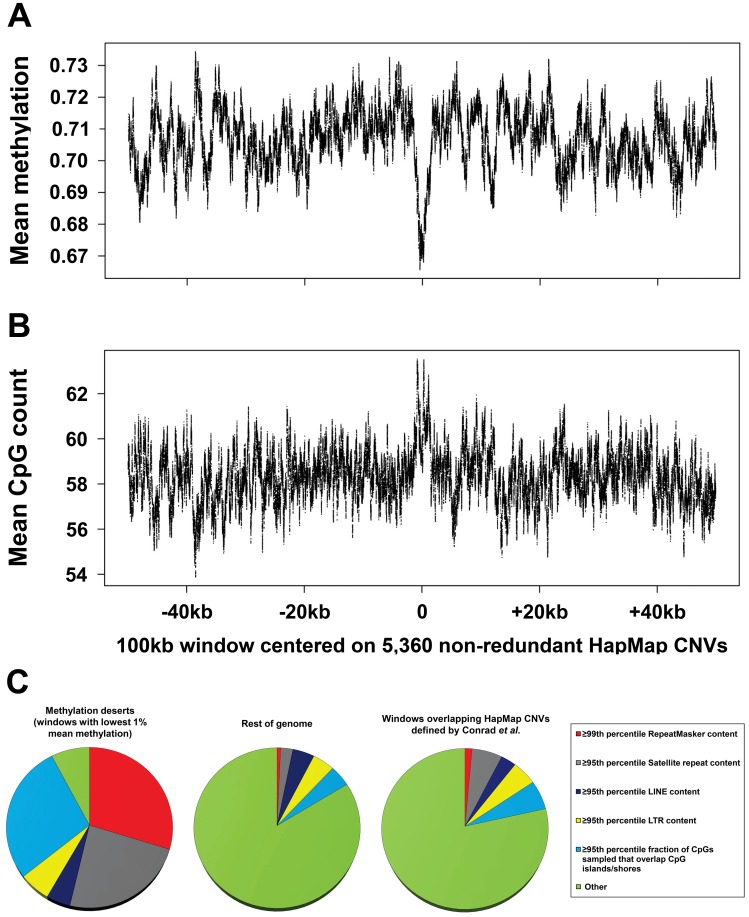
Global assessment of methylation levels and confounders contributing to hypomethylation in common CNV regions. (a) Mean methylation levels and (b) mean CpG density per base within and flanking 5,360 nonredundant HapMap CNVs. To directly assess the relationship between DNA methylation and structural variation, we used published 15× bisulfite sequencing data [10] to calculate mean methylation per base both within and flanking a high-quality set of HapMap CNVs [7]. We first merged 8,599 CNVs defined by Conrad into 6,142 nonredundant regions, and then removed those <20 kb in size to form a filtered set of 5,360 nonredundant regions (mean size, 3,789 bp). A 100 kb window was then centered on the midpoint of each CNV, and mean methylation levels and CpG count per base in these 100 kb windows were calculated using 15× sperm bisulfite sequencing data [10]. Each plot shows a 100 bp moving average. Although a small decrease in methylation level is evident within CNVs compared to flanking regions, overall mean methylation levels within CNV regions (69%) are very similar to the genome average (70%). Furthermore this dip in methylation corresponds precisely with an increase in CpG density and an enrichment for CGIs within CNVs. As most CGIs are unmethylated in sperm [10], [17], this fact likely accounts for the small overall reduction in methylation levels associated with CNVs. (c) Regions classified as “methylation deserts” by Li et al. represent an extremely nonrandom subset of the genome that is highly enriched for common repeats and preferential mapping of bisulfite reads to CpG islands. We classified all 100 kb windows defined by Li et al. based on their content of common repeats and fraction of CpGs assayed that map within ±2 kb of CGIs. One hundred and eighty-three of the 285 (64%) windows that were classified as “methylation deserts” by Li et al. are >95^th^ percentile based on satellite, LINE, or LTR content and/or the 99^th^ percentile based on total repeat content. A further 80 windows (28%) are >95^th^ percentile based on the fraction of CpGs assayed within them that map to CGIs or shores. Overall, only 22 of 285 (8%) windows defined by Li et al. as “methylation deserts” do not show extremes of repeat content or highly biased sampling of CpG islands. In contrast, in the rest of the genome, 84% of windows do not overlap any of these categories. Furthermore, windows that overlap a high-quality dataset of HapMap CNVs [7] show a repeat content and proportion of reads mapping to CGIs similar to the genome average. Thus, the set of regions defined as “methylation deserts” by Li et al. represent an extreme fraction of the genome that is likely to be highly enriched for unusual epigenetic and structural features.

In summary, we identify multiple strong confounders in the study of Li et al. that in our opinion cast serious doubt on the notion that germline hypomethylation is causally related to structural mutability. Overall, our analysis shows that 92% of the regions defined as “methylation deserts” by Li et al. are composed of extremely high repeat content or show preferential sampling of unmethylated CpG islands (Figure 2c). After removing these biases we fail to observe any enrichment for CNVs in hypomethylated regions of the genome (Figure 1b). Although the question of whether epigenetic variation plays a role in structural mutability remains open, we caution that in order to maintain robustness any future studies must carefully control for numerous potential confounders associated with CNVs.

## Supporting Information

Figure S1 Regions of the genome with low mean methylation show a reduction in the proportion of CpG dinucleotides with at least one overlapping read. Box plots show the fraction of CpG dinucleotides within each 100 kb window that are covered by at least one overlapping bisulfite read.
(TIF)

Figure S2 An example window from the Li et al. study (chr15:80596736–80616700). This window was scored as a “methylation desert” (within the bottom 1% of mean methylation in the genome). However, this region contains no SNPs, and only 27/355 (7.6%) of the CpG dinucleotides in this region have at least one overlapping bisulfite read. Of the 27 CpG sites assayed, 21 (78%) lie within a CpG island that spans the promoter of *RPS17*, a gene expressed in testes. While all sites sampled within this CpG island show low methylation (<25%), most CpGs sampled in the rest of the window have high methylation (>75%), suggesting that the low mean methylation level in this window is biased due to preferential sampling of sites within the CpG island. The low frequency of HapMap SNPs and uniquely mappable bisulfite reads in this region is attributable to the presence of a segmental duplication of 99.85% identity that is also copy number variant. Screenshot taken from the UCSC Genome Browser. Scatter plot shows methylation levels at the 27 CpGs assayed (blue circles/dotted line) and the location of the 328 other CpGs (grey crosses), which have no overlapping reads.
(TIF)

Figure S3 Regions of the genome in which bisulfite reads map preferentially to CpG islands/shores are enriched for structural variation, segmental duplications, and genes highly expressed in testes. Using the same set of 100 kb windows as Li et al., we first excluded any window containing satellite repeats, or those >99^th^ percentile based on their content of LINEs, SINEs, LTRs, or total repeats. Bar plots show mean values for all windows in the genome, and in windows >95^th^ or >99^th^ percentiles based on the fraction of CpGs assayed within each window that mapped within ±2 kb of CpG islands. We observed large enrichments for overlaps with multiple CNV datasets, segmental duplications, and genes that show high relative expression in testes (defined here as >5-fold higher expression in human testes versus the mean of five other tissues) [18]. These relationships create a strong confounder that results in regions that were scored as hypomethylated based on mean methylation level having a strong bias to also be scored as structurally variant. In contrast, we observed that mean methylation levels outside CpG islands/shore regions are consistently high in all categories.
(TIF)

Figure S4 Power calculations showing the probability of observing at least one mSNP per window as a function of total SNP content. Based on the relative prevalence of mSNPs (0.08163) and non-mSNPs (0.91837) among all HapMap SNPs in the genome, and presuming that mSNPs are randomly distributed among all SNPs, the probability of observing at least one mSNP in any given window is given by the formula *p* = 1-(0.91837)^n^, where n is the number of SNPs per window. Based on this calculation, a minimum sample size of 28 SNPs is required per window to provide >90% probability of observing at least one mSNP by chance, corresponding to a false discovery rate for regions with MI = 0 simply due to insufficient sample size of <10%. Dashed lines show the median SNP number in windows with MI = 0 compared to that in the whole genome.
(TIF)

Table S1 Annotation of 28,441 windows of the human genome used in the study of Li et al. Data were obtained from the UCSC Genome Browser (hg18 assembly, http://genome.ucsc.edu/) or from published studies [7], [10], [12]–[14], [18], with intersections, and where necessary liftovers between genome assemblies, performed using Galaxy (https://main.g2.bx.psu.edu/).
(XLSX)
