# Supplementary figures and images for: Comment on “Genomic Hypomethylation in the Human Germline Associates with Selective Structural Mutability in the Human Genome”
Source: PLoS Genet. 2013 Feb 28;9(2):e1003332. doi: 10.1371/journal.pgen.1003332 (PMC3585013; doi:10.1371/journal.pgen.1003332)

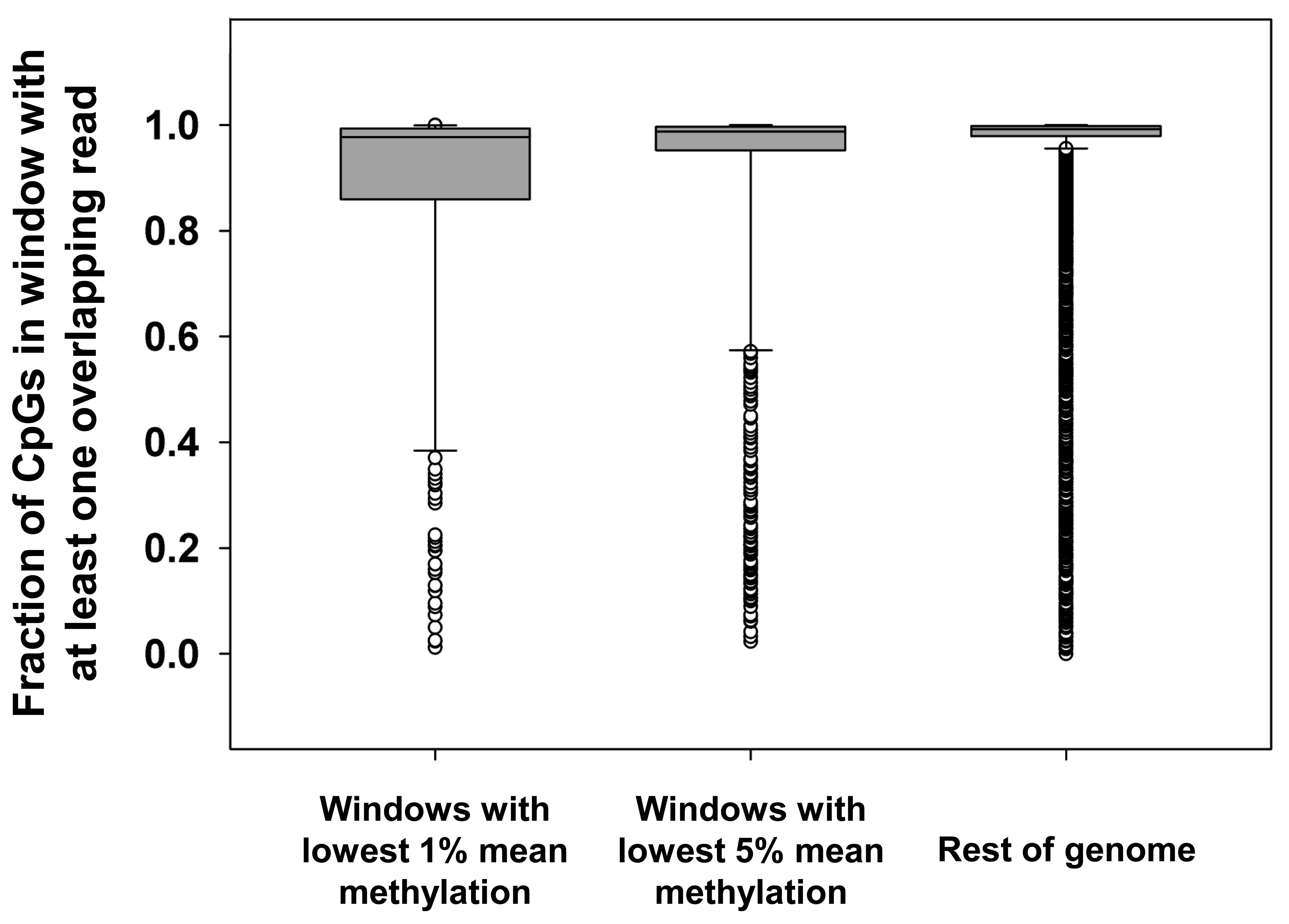

Supplement: Figure S1 — Regions of the genome with low mean methylation show a reduction in the proportion of CpG dinucleotides with at least one overlapping read. Box plots show the fraction of CpG dinucleotides within each 100 kb window that are covered by at least one overlapping bisulfite read. (TIF) [file pgen.1003332.s001.tif]

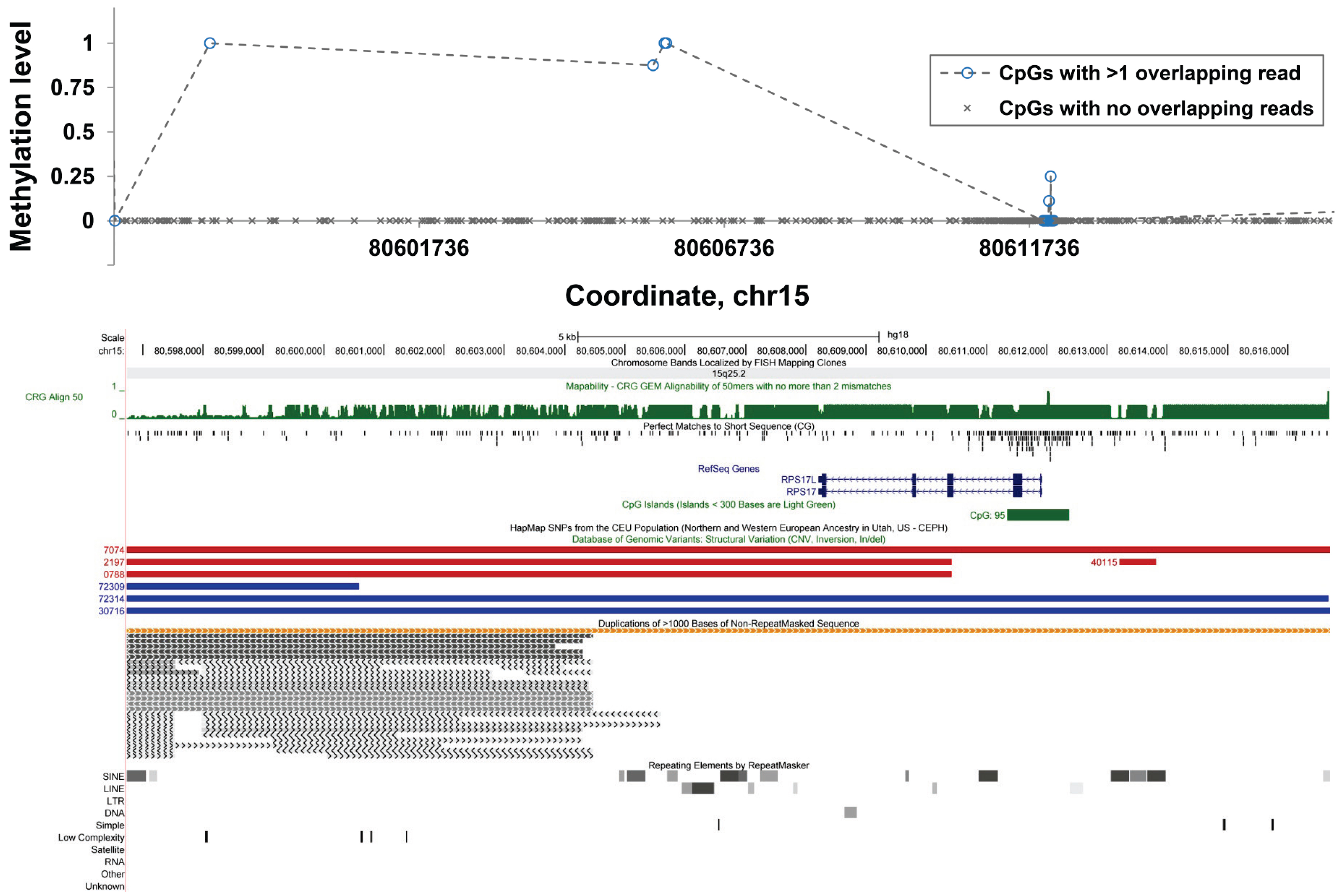

Supplement: Figure S2 — An example window from the Li et al. study (chr15:80596736–80616700). This window was scored as a “methylation desert” (within the bottom 1% of mean methylation in the genome). However, this region contains no SNPs, and only 27/355 (7.6%) of the CpG dinucleotides in this region have at least one overlapping bisulfite read. Of the 27 CpG sites assayed, 21 (78%) lie within a CpG island that spans the promoter of RPS17, a gene expressed in testes. While all sites sampled within this CpG island show low methylation (<25%), most CpGs sampled in the rest of the window have high methylation (>75%), suggesting that the low mean methylation level in this window is biased due to preferential sampling of sites within the CpG island. The low frequency of HapMap SNPs and uniquely mappable bisulfite reads in this region is attributable to the presence of a segmental duplication of 99.85% identity that is also copy number variant. Screenshot taken from the UCSC Genome Browser. Scatter plot shows methylation levels at the 27 CpGs assayed (blue circles/dotted line) and the location of the 328 other CpGs (grey crosses), which have no overlapping reads. (TIF) [file pgen.1003332.s002.tif]

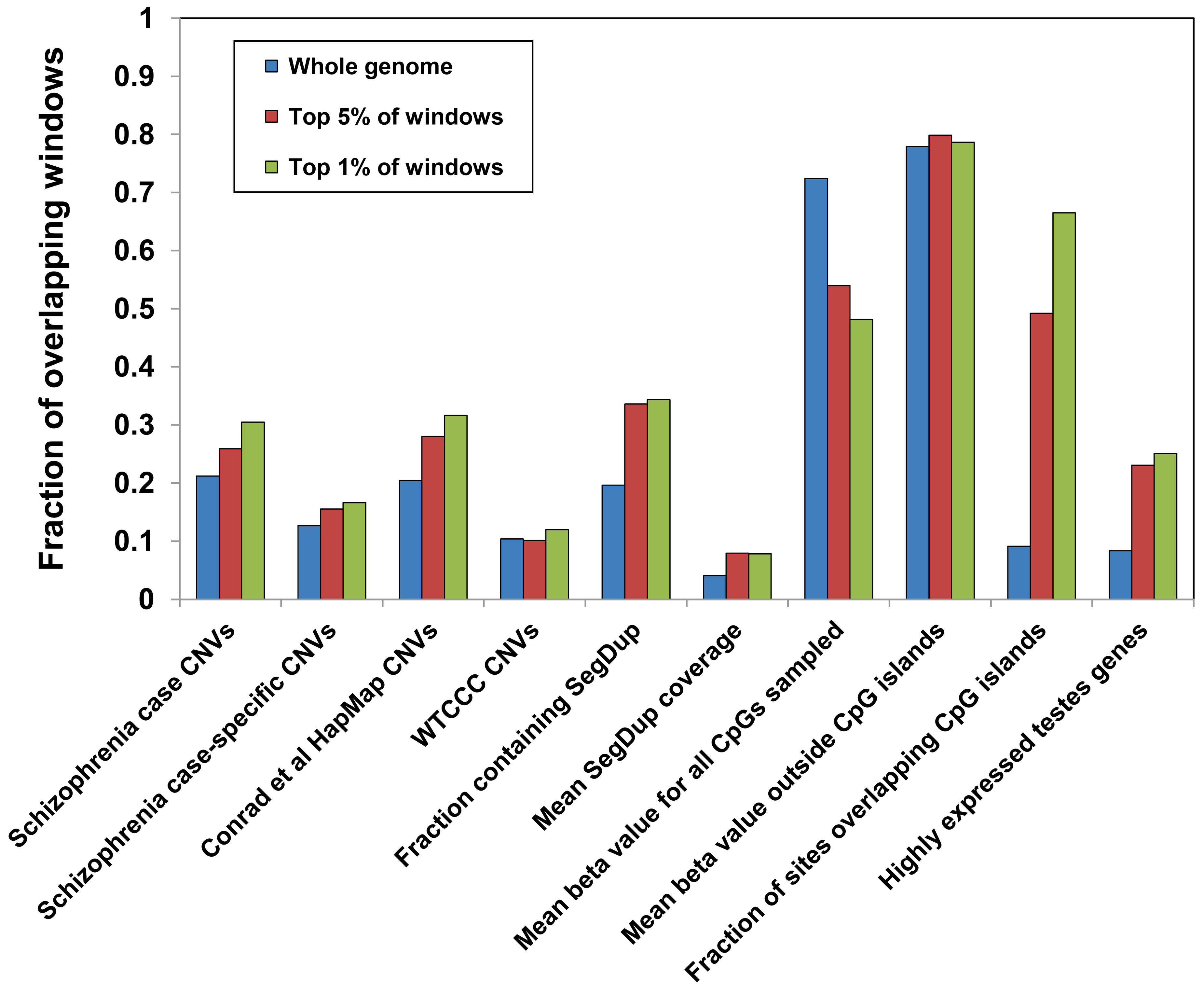

Supplement: Figure S3 — Regions of the genome in which bisulfite reads map preferentially to CpG islands/shores are enriched for structural variation, segmental duplications, and genes highly expressed in testes. Using the same set of 100 kb windows as Li et al., we first excluded any window containing satellite repeats, or those >99th percentile based on their content of LINEs, SINEs, LTRs, or total repeats. Bar plots show mean values for all windows in the genome, and in windows >95th or >99th percentiles based on the fraction of CpGs assayed within each window that mapped within ±2 kb of CpG islands. We observed large enrichments for overlaps with multiple CNV datasets, segmental duplications, and genes that show high relative expression in testes (defined here as >5-fold higher expression in human testes versus the mean of five other tissues) [18]. These relationships create a strong confounder that results in regions that were scored as hypomethylated based on mean methylation level having a strong bias to also be scored as structurally variant. In contrast, we observed that mean methylation levels outside CpG islands/shore regions are consistently high in all categories. (TIF) [file pgen.1003332.s003.tif]

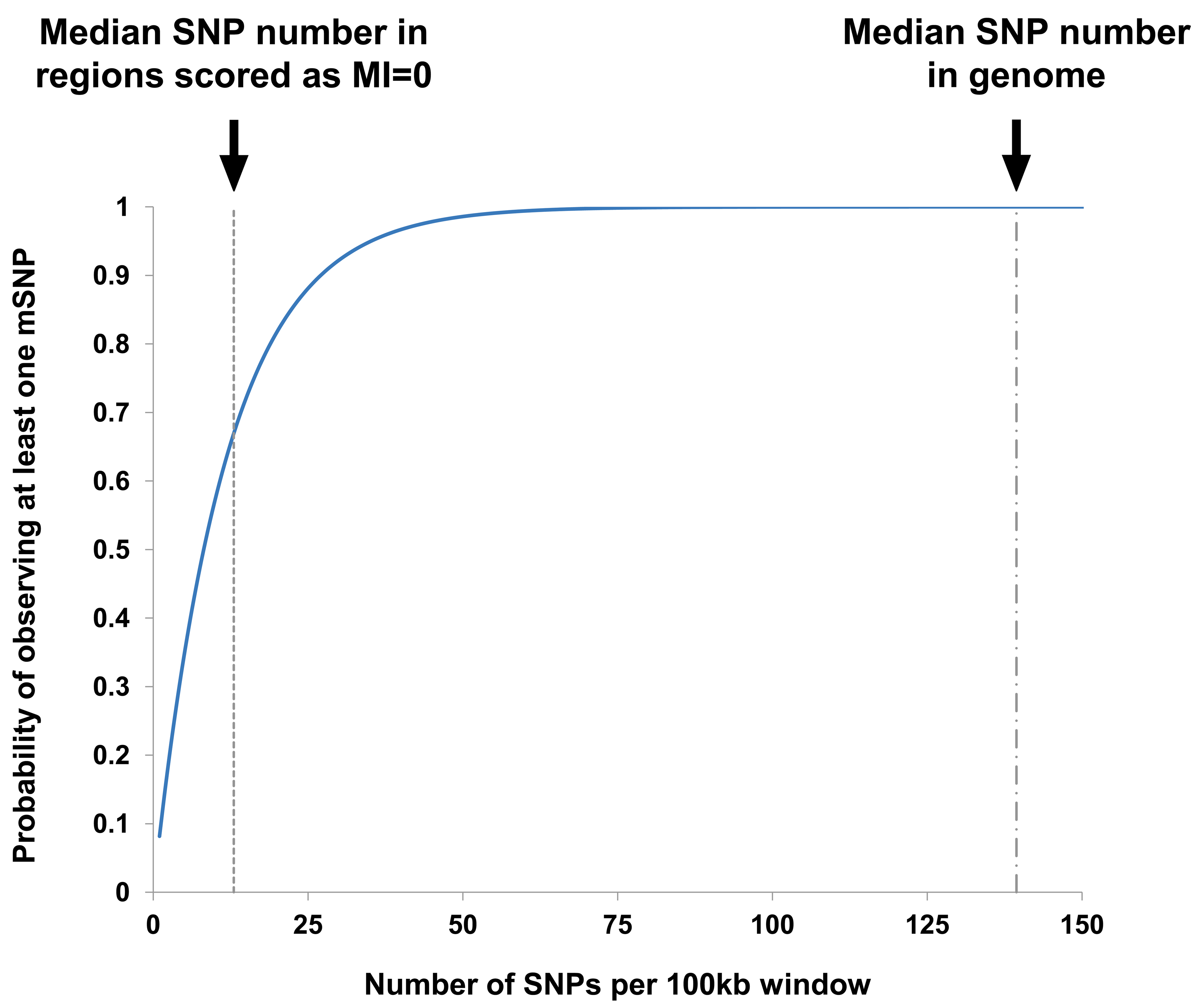

Supplement: Figure S4 — Power calculations showing the probability of observing at least one mSNP per window as a function of total SNP content. Based on the relative prevalence of mSNPs (0.08163) and non-mSNPs (0.91837) among all HapMap SNPs in the genome, and presuming that mSNPs are randomly distributed among all SNPs, the probability of observing at least one mSNP in any given window is given by the formula p = 1-(0.91837)n, where n is the number of SNPs per window. Based on this calculation, a minimum sample size of 28 SNPs is required per window to provide >90% probability of observing at least one mSNP by chance, corresponding to a false discovery rate for regions with MI = 0 simply due to insufficient sample size of <10%. Dashed lines show the median SNP number in windows with MI = 0 compared to that in the whole genome. (TIF) [file pgen.1003332.s004.tif]
